# Supplementary material for: Cancer-Associated Thrombosis on Bevacizumab: Risk of Recurrence and Bleeding When Bevacizumab Is Stopped or Continued
Source: Cancers (Basel). 2023 Jul 31;15(15):3893. doi: 10.3390/cancers15153893 (PMC10417508; doi:10.3390/cancers15153893)
Supplement: Supplementary file 1 [file cancers-15-03893-s001.zip › cancers-2480154-supplementary.pdf]

Supplementary Material

Screening methodology of the patients in the three centers.

At the Georges Pompidou European Hospital: the term "bevacizumab" or "Avastin" was searched in the DX Care® computerized records as well as the PMSI VTE or PE coding (I26 or I80) between June 2000 and December 2020. Data were collected from the DX Care® software. Treatment periods were manually verified using data extracted from the CHIMIO® software.

At Louis Mourier Hospital: the list of patients who received bevacizumab / Avastin® was extracted from the prescription software between April 2009 and December 2020. The occurrence of VTE during the bevacizumab treatment period and data were collected from the Orbis® software. Treatment periods were manually verified using CHIMIO® software.

At the Georges François Leclerc Center: the terms "pulmonary embolism" or "venous thrombosis" were searched in the reports as well as the prescription of chemotherapy by bevacizumab / Avastin® on the Consore® software between June 2008 and December 2020. The occurrence of a VTE during the bevacizumab treatment period was manually checked for each record in Consore® and Clinicom®. Data and treatment periods were collected on Clinicom®.

**Cancer type**	**N (%)**
Cancer type<br>Colorectal	78 (48)
Ovarian, endometrial	28 (17)
Breast	15 (9)
Lung	18 (11)
Central nervous system	17 (11)
Liver cancer	1 (0.6)
Undifferentiated carcinoma pancreatic cancer	1 (0.6)
Neuroendocrine carcinoma of unknown primary site	1 (0.6)
Biliary cancer	1 (0.6)
Kidney cancer	1 (0.6)
Pancreatic cancer	1 (0.6)

Table S1. Cancer subtypes. Variables are expressed as absolute value (percentage).

	**Population n = 162**	**No VTE recurrence nor bleeding n=114**	**VTE recurrence or bleeding n = 48**	**p-value**
Age at diagnosis	64 [55–71]	64 [55–71]	63 [54–72]	0.91
Sex				
Female	96 (59%)	67 (59%)	29 (60%)	0.86
Male	66 (41%)	47 (41%)	19 (40%)	
BMI (n=156)	24.6 [21.0–28.0]	23.1 [20.4–27.0]	26.0 [23.0–28.2]	**0.01**
ECOG Performance Status				
0-1	130/153 (85%)	89/107 (83%)	41/46 (89%)	0.45
2-3	23 (15%)	18 (17%)	5 (11%)	
	27/144 (19%)	21/102 (21%)	6/42(14%)	0.48
Renal failure *(eGFR < 60 mL/mn)*				
Anemia *(Hb<100 g/L)*	11/153 (7%)	10/110 (9%)	1/43 (2%)	0.18
Thrombocytosis *(>300G/L)*	38/148 (26%)	27/106 (25%)	11/42 (26%)	> 0.99
Antiaplatelet therapy	17/154 (11%)	11/91(12%)	6/63 (10%)	0.79
Cancer type				
Colorectal	78 (48%)	56 (49%)	22 (46%)	0.73
Ovarian, endometrial	28 (17%)	21 (18%)	7 (15%)	0.65
Breast	15 (9%)	10 (9%)	5 (10%)	0.77
Lung	18 (11%)	10 (9%)	8 (17%)	0.17
Central nervous system	17 (10%)	13 (11%)	4 (8%)	0.78
Others	6 (4%)	4 (4%)	2 (4%)	> 0.99
Histological subtype				
Adenocarcinoma	119 (73%)	83 (73%)	36 (75%)	0.85
Current treatment line				
1st line	74/152 (49%)	50/105 (48%)	24/47 (51%)	0.73
2nd line	50 (33%)	34 (32%)	16 (34%)	0.85
3rd line	28 (18%)	21 (20%)	7 (15%)	0.51
Platinum salt treatment	62 (38%)	45 (39%)	17 (35%)	0.72
Metastatic disease	123/146 (84%)	88/102 (86%)	35/44 (80%)	0.33
Metastases				
Cerebral	9 (6%)	5 (4%)	4 (8%)	0.45
Hepatic	57 (35%)	39 (34%)	18 (38%)	0.72

VTE : venous thromboembolic events, BMI: body mass index, eGFR: estimated glomerular filtration rate, Hb: hemoglobin.

Table S2. Baseline demographic and oncologic characteristics according to the occurrence of the primary endpoint. Variables are expressed as median [interquartile range], or absolute value (percentage). Statistically significant values are in bold.

	Population n = 162	No VTE recurrence nor bleeding n=114	VTE recurrence nor bleeding n = 48	p-value
CAT localization				
DVT	58 (36%)	41 (36%)	17 (35%)	0.99
PE	81 (50%)	55 (48%)	26 (54%)	0.61
DVT and PE	23 (14%)	18 (16%)	5 (20%)	0.46
PE localization				
Segmental or more proximal	79/97 (81%)	52/67 (77%)	27/30 (90%)	0.17
Subsegmental	18 (19%)	15/67	3/30 (10%)	
Unilateral	55/98 (56%)	39/68 (57%)	16/30 (53%)	0.38
Bilateral	43 (44%)	29 (43%)	14 (47%)	
Discovery mode				
Clinically suspected	82/160 (51%)	60/113 (53%)	22/47 (47%)	0.49
Incidental asymptomatic	14 (9%)	9 (8%)	5 (11%)	0.60
Incidental symptomatic	72 (45%)	49 (43%)	23 (49%)	0.39
Time between CAT and first inclusion (days)	3080 [1947–4089]	2991 [1869–4045]	239 [2587–4121]	0.15
LMWH therapy	152 (94%)	92 (94%)	59 (94%)	> 0.99
Time between bevacizumab initiation and CAT (days)	79 [39–154]	78 [39–153]	82 [47–150]	0.84
Bevacizumab posology at CAT diagnosis (mg/kg) (n=135)	7.5 [5–10]	7.5 [5–10]	7.5 [5–10]	0.88
Platinum salt treatment	62 (38%)	45 (39%)	17 (35%)	0.72
Other risk factor of CAT	37 (23%)	27 (24%)	10 (21%)	0.84
Response to oncologic treatment				
Response	40/154 (26%)	24/107(22%)	16/47 (34%)	0.16
Stability	76 (49%)	55 (51%)	21 (45%)	0.49
Progression	38 (25%)	28 (26%)	10 (21%)	0.55

CAT: cancer associated thrombosis, DVT: deep veinous thrombosis, PE: pulmonary embolism LMWH: low molecular weight heparin

Table S3. Initial thromboembolism characteristics according to the occurrence of the primary endpoint. Variables are expressed as median [interquartile range], or absolute value (percentage). Statistically significant values are in bold.

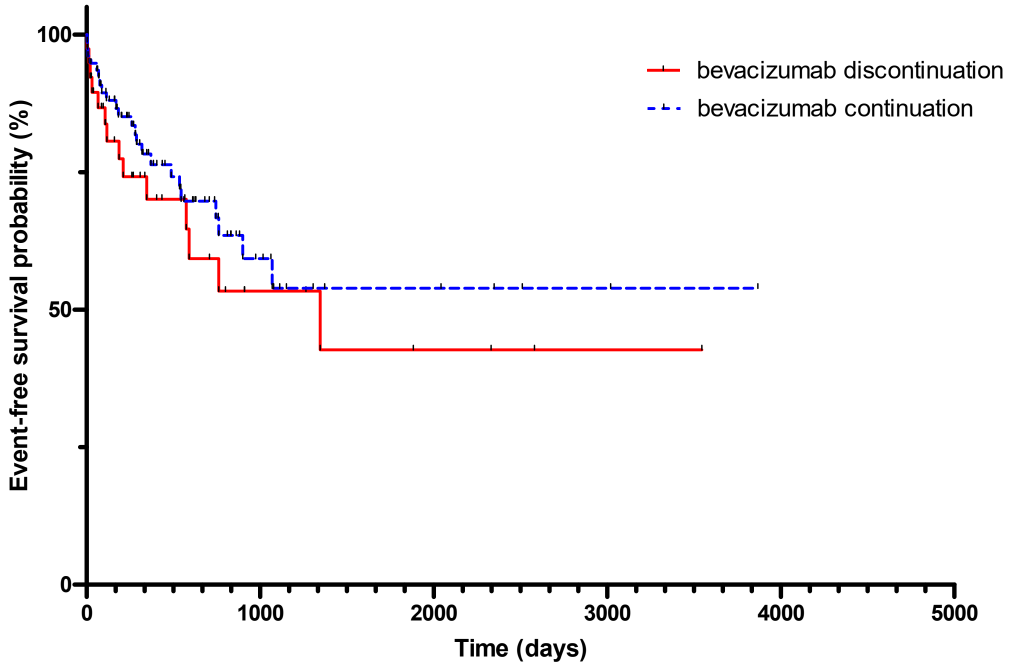

Figure S1. Subgroup analysis in patient with a tumoral response or stability at the time of the CAT: occurrence of recurrent cancer associated thrombosis or bleeding according to the continuation or discontinuation of bevacizumab. Hazard Ratio 0.76 for continuation, 95%CI (0.38–1.51), p=0.355.

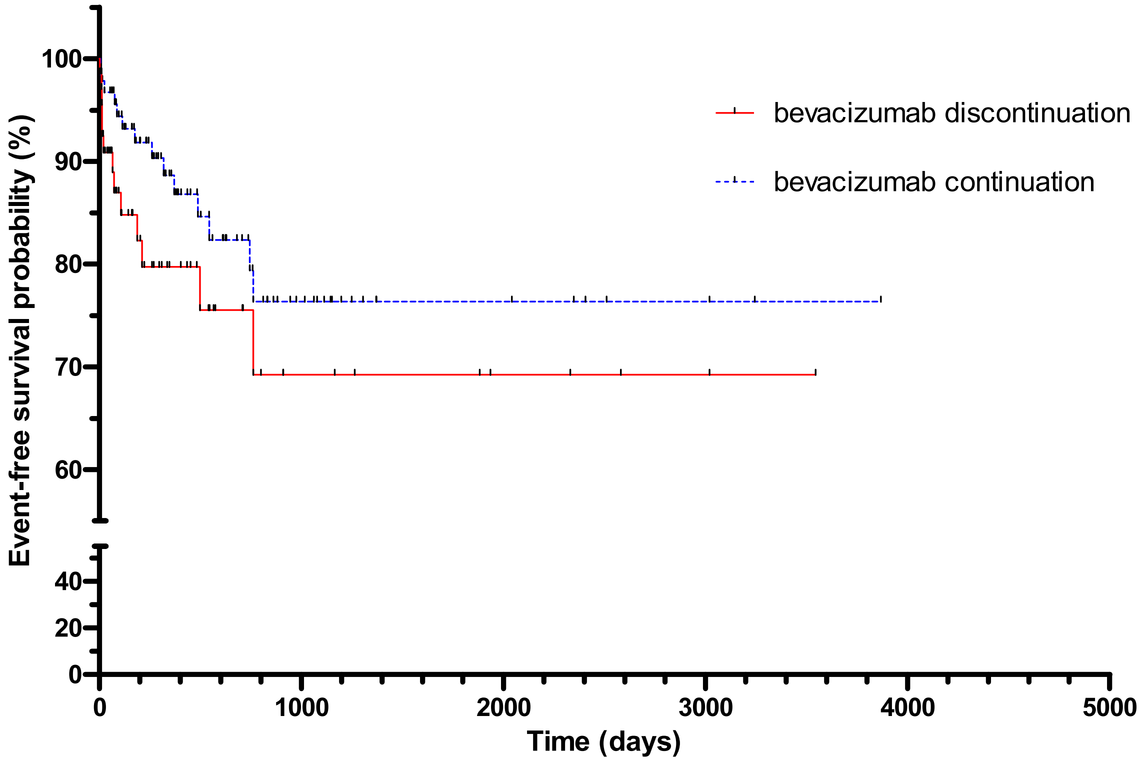

Figure S2. Bleeding according to continuation or discontinuation of bevacizumab. Hazard Ratio 0.64 for continuation, 95%CI (0.29-1.38) p = 0.225.

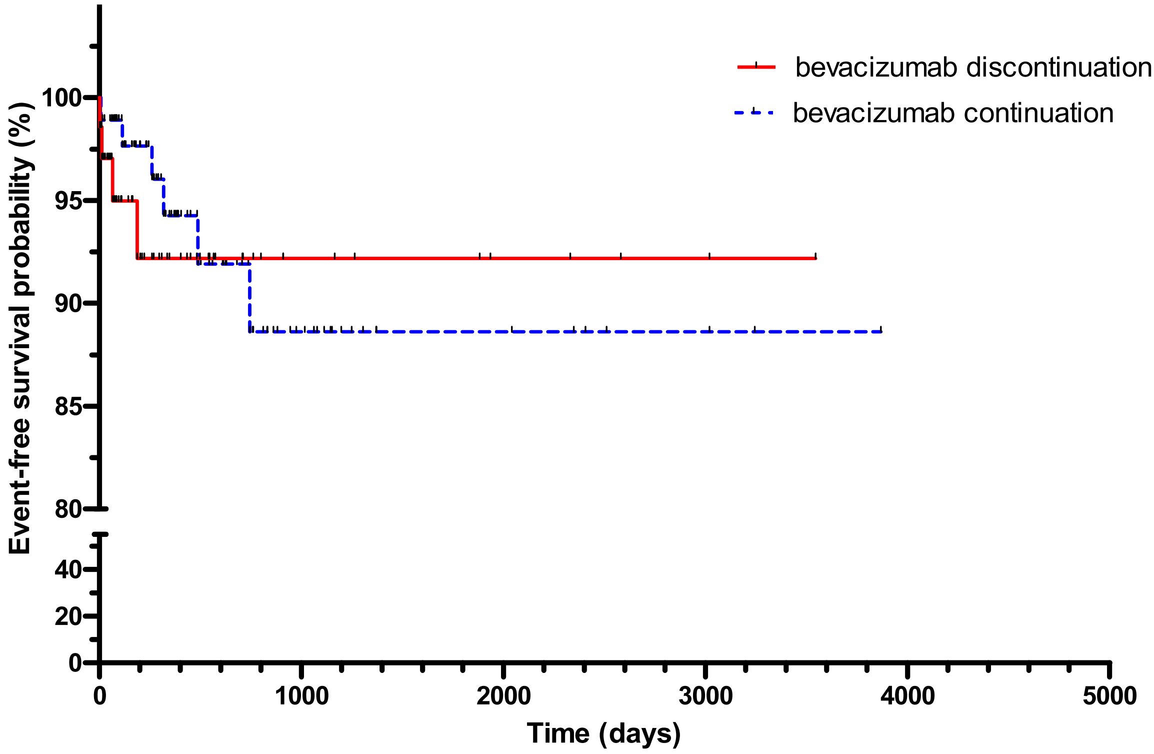

Figure S3. Major bleeding according to continuation or discontinuation of bevacizumab. Hazard Ratio 0.83 for continuation, 95%CI (0.23–3.02), p=0.766.

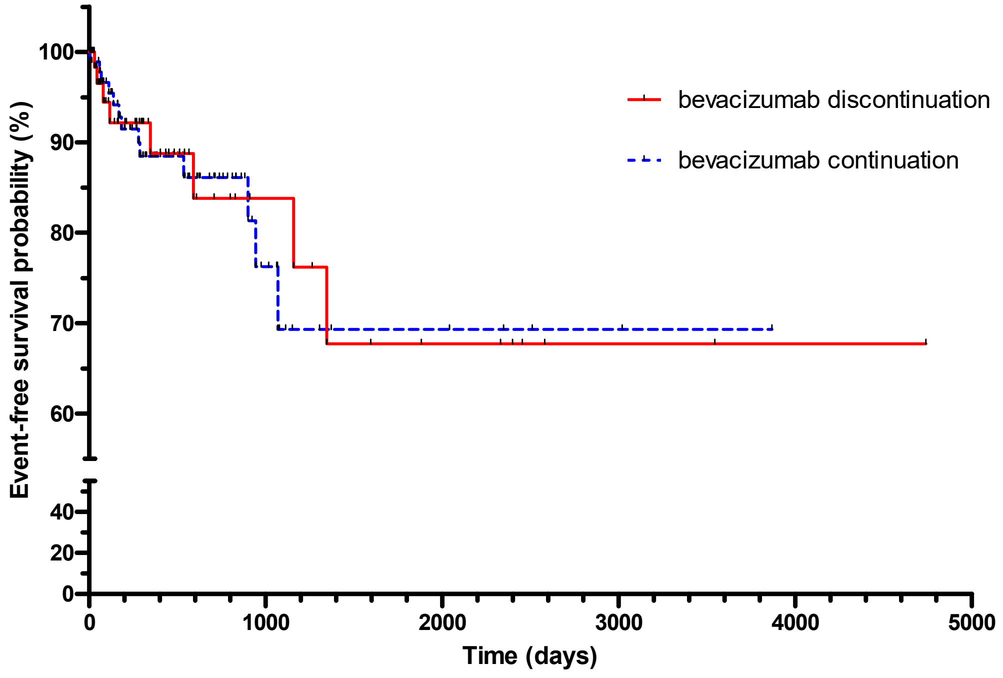

Figure S4. **Recurrence of CAT according to continuation or discontinuation of bevacizumab**. Hazard Ratio 0.93 for continuation, 95%CI (0.40-2.19) p=0.869.
